# Case report: Ultrasound and contrast-enhanced ultrasound findings of pediatric small intestinal inflammatory myofibroblastic tumor

**DOI:** 10.3389/fonc.2025.1512402

**Published:** 2025-02-06

**Authors:** Zengmiao Xing, Tangna Wu, Lingling Qin

**Affiliations:** Department of Ultrasonography, Hainan General Hospital (Hainan Affiliated Hospital of Hainan Medical University), Haikou, Hainan, China

**Keywords:** pediatric, inflammatory myofibroblastic tumor, small intestine, ultrasound, contrast enhanced ultrasound

## Abstract

Inflammatory myofibroblastic tumors (IMTs) are uncommon mesenchymal neoplasms with malignant potential, primarily affecting children and adolescents. It usually manifests in the abdominal and pelvic regions; however, small intestinal IMT is particularly rare. This report presents the case of a 7-year-old girl who presented with a small intestinal IMT. The patient was admitted with a one-day history of abdominal pain and vomiting. Ultrasonography revealed a solid hypoechoic mass in the lower abdomen. Based on contrast-enhanced ultrasound (CEUS) findings, a preliminary diagnosis of small intestinal IMT was proposed, which was subsequently confirmed by postoperative histopathology. This case underscores the sonographic and CEUS features of small intestinal IMT in children, emphasizing that the combination of ultrasound and CEUS can improve diagnostic accuracy and preoperative evaluation in pediatric patients.

## Introduction

Inflammatory myofibroblastic tumors (IMTs) are rare mesenchymal tumors with malignant potential. They mainly consist of differentiated myofibroblastic spindle cells and are often accompanied by inflammatory cells, including plasma cells, lymphocytes, and eosinophils (1). IMT is commonly observed in both children and adolescents. They can occur in various organs throughout the body (2, 3). However, IMT in the small intestine in children is particularly rare (3, 4). The varied clinical presentations of IMT and its non-specific imaging characteristics often lead to misdiagnosis (5). Ultrasound and contrast-enhanced ultrasound (CEUS) have demonstrated particular advantages in the differential diagnosis of small intestinal tumors in pediatric patients. This report presents a case of small intestinal IMT in a 7-year-old girl, initially diagnosed through preoperative ultrasound combined with CEUS and subsequently confirmed by postoperative pathology. The case highlights the ultrasound and CEUS characteristics of pediatric small intestinal IMT.

## Case presentation

A 7-year-old girl presented with a one-day history of vomiting and abdominal pain. The patient had no significant medical history. Physical examination revealed marked tenderness in the mid-and lower abdomen without rebound tenderness. A palpable mass, approximately 6.0 × 4.0 cm in size, with a firm consistency and clear boundaries, was detected in the lower abdomen. Bowel sounds were normal.

Laboratory tests showed a normal white blood cell count (WBC; 10.5 × 10^9^/L; reference range: 4.3-11.3 × 10^9^/L), slightly elevated neutrophil percentage (NE; 80.6%; reference range: 31-70%), and increased C-reactive protein (CRP; 58.33 mg/L; reference range: 0-8 mg/L). Mild microcytic hypochromic anemia was present, with a hemoglobin level of 107 g/L (reference range: 118-156 g/L), mean corpuscular volume (MCV) of 75.2 fL (reference range: 77-92 fL), and mean corpuscular hemoglobin (MCH) of 24.4 pg (reference range: 25-34 pg). The platelet count was slightly elevated (536 × 10^9^/L; reference range:167-453 × 10^9^/L). Urinalysis, stool analysis, liver and kidney function tests, and tumor marker (AFP, CEA, CA125, NSE, CA19-9, CA242, CA15-3, ferritin, HCG, and growth hormone) were all within normal limits.

**Figure 1:**
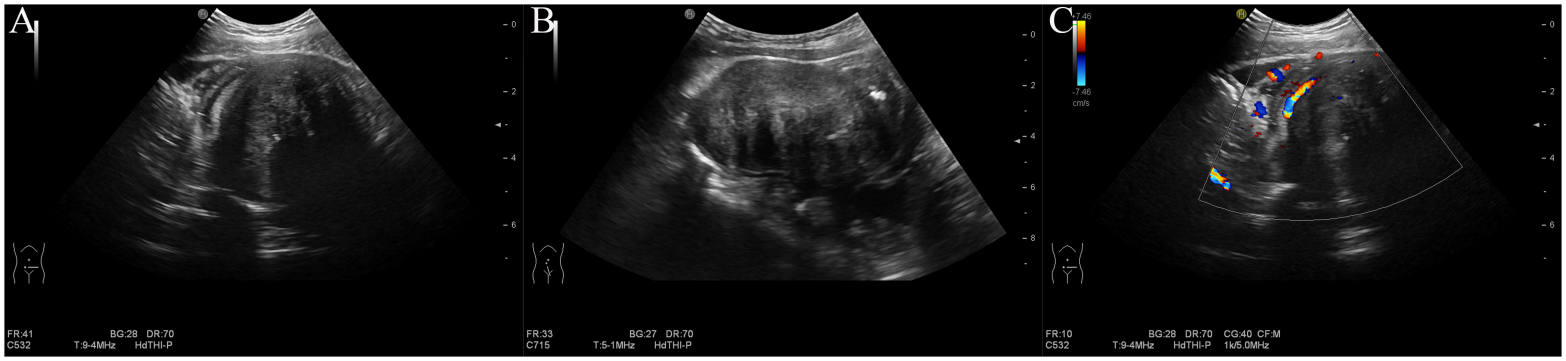
Conventional ultrasound. **(A)** Transverse view: a hypoechoic mass in the lower abdomen with slightly hyperechoic fibrous strands and attenuation. **(B)** Longitudinal view of the mass. **(C)** Color doppler imaging reveals blood flow signals within and surrounding the mass, originating from the small intestine wall and mesentery.

Abdominal ultrasonography revealed a solid hypoechoic mass in the lower abdominal cavity measuring approximately 8.1 × 4.8 × 5.0 cm. The mass had a well-defined and smooth border with heterogeneous internal echoes and featured fibrous strands with slightly higher echoes and central echo attenuation. Mild calcification was observed in the mass. The mass was contiguous with the wall of the small intestine and partially encircled it. Proximal dilation of the small intestine was noted, with a maximum width of approximately 3.0 cm. The intestinal wall was slightly thickened, with normal peristalsis. Color Doppler Flow Imaging (CDFI) revealed a small amount of signal within the mass, with linear blood flow signals observed at the periphery originating from the surrounding small intestinal wall and mesentery (**Figure 1**). Ultrasonography suggested that the mass was likely a small intestinal tumor, with a potential diagnosis of inflammatory myofibroblastic tumor, and was associated with partial bowel obstruction. Other differential diagnoses to consider included small bowel lymphoma and gastrointestinal stromal tumor, both of which can present with similar clinical and imaging features. CEUS was recommended for further evaluation.

**Figure 2:**
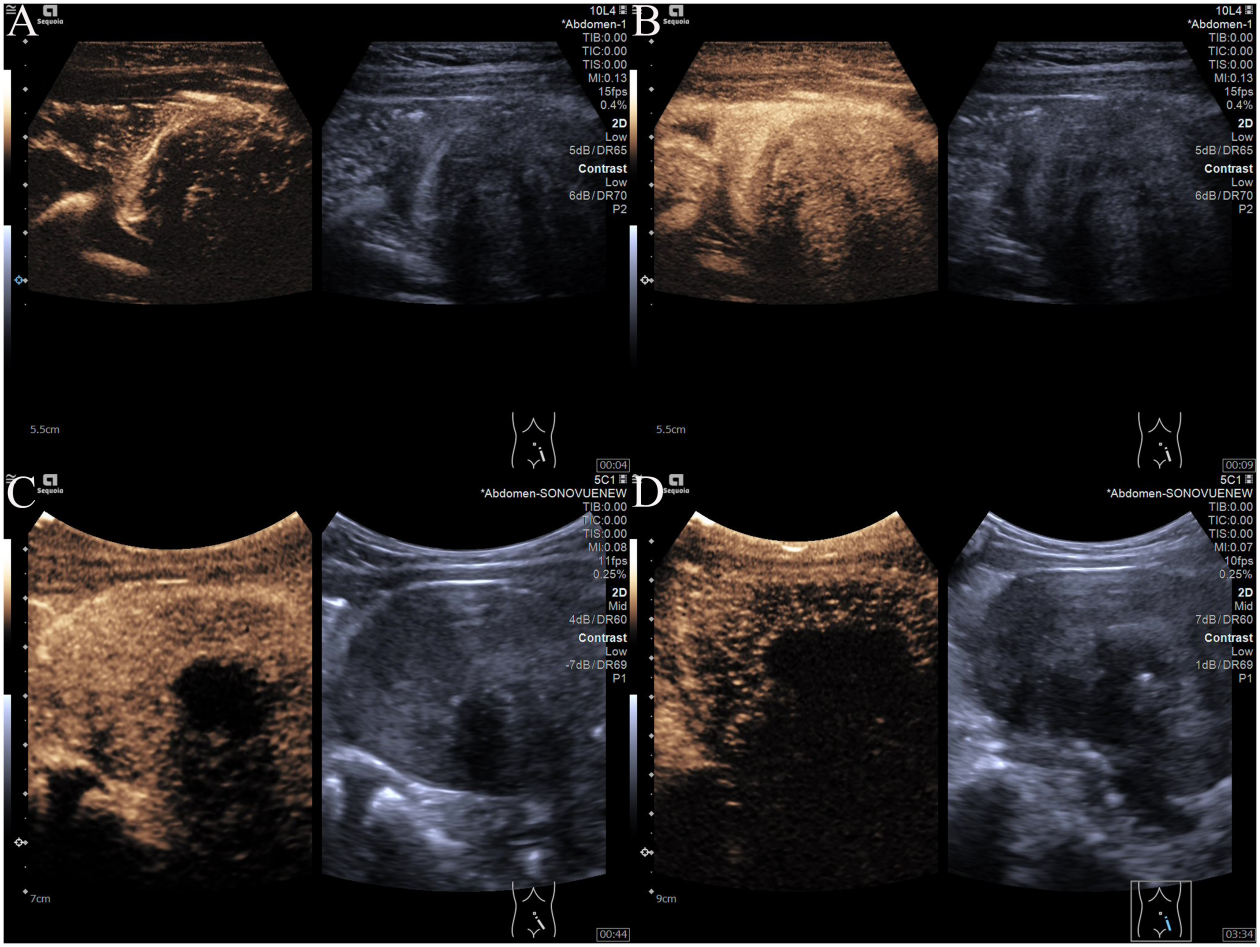
Contrast-enhanced ultrasound. **(A)** Arterial phase at 4 seconds: peripheral enhancement observed. **(B)** Peak enhancement at 9 seconds: slight hypo-enhancement detected. **(C)** Venous phase: hypo-enhancement with a central non-perfused area. **(D)** Enhancement persisting up to 3.5 minutes.

After informed consent was obtained from the guardians, CEUS was performed. Following the intravenous injection of 2 ml SonoVue contrast agent (Bracco), 5 ml of saline was injected for flushing. CEUS revealed synchronous enhancement of the mass and the surrounding bowel wall during the arterial phase, starting approximately 4 seconds after injection (**Figure 2A**). The mass exhibited a slightly lower enhancement, peaking at approximately 9 seconds, followed by slow washout and marginal peripheral enhancement (**Figure 2B**). Peripheral enhancement is also observed. In the venous phase, persistently low enhancement was noted (**Figure 2C**), with residual enhancement within the lesion persisting for up to 3.5 minutes (**Figure 2D**). The echo attenuation zone observed on conventional ultrasound was not perfused by the contrast agent. Based on the CEUS characteristics, an IMT of the small intestine secondary to partial small bowel obstruction was considered.

**Figure 3:**
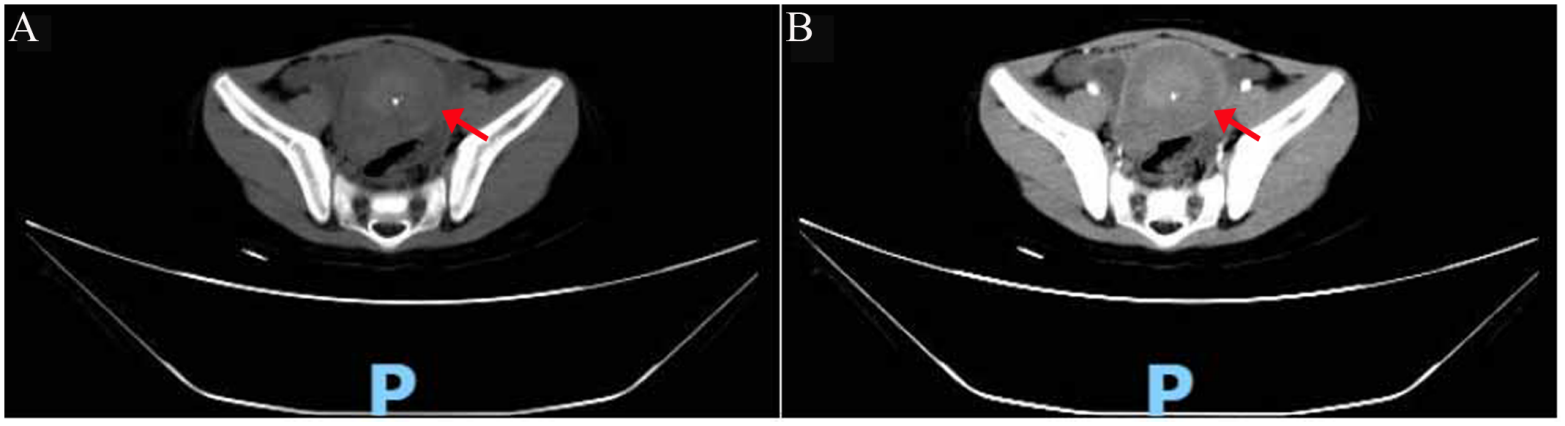
CT scans. **(A)** Plain CT: mixed-density mass in the abdominopelvic cavity (red arrow). **(B)** Contrast-enhanced CT: heterogeneous and peripheral enhancement (red arrow). CT, computed tomography.

Non-contrast computed tomography (CT) revealed a mixed-density mass in the pelvic cavity with internal calcifications (**Figure 3A**). Contrast-enhanced CT revealed heterogeneous enhancement of the mass, with significant peripheral enhancement and continuous enhancement of the small intestinal wall surrounded by the adjacent mesentery (**Figure 3B**).

**Figure 4:**
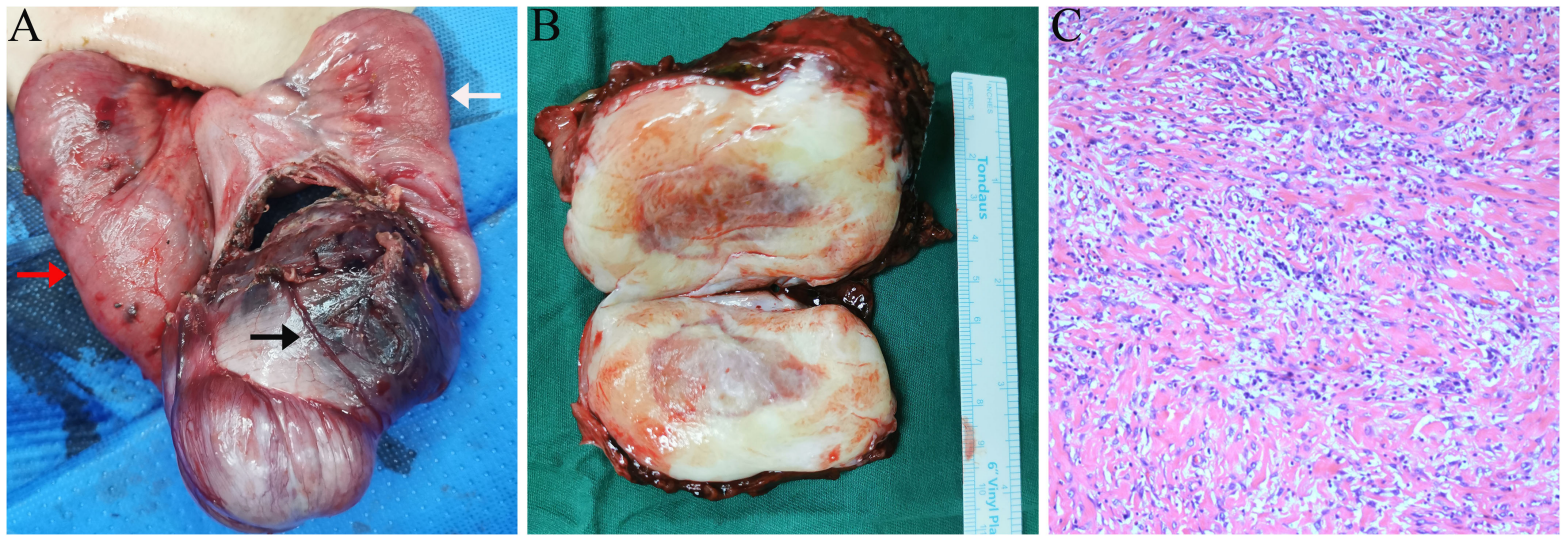
Intraoperative and pathology findings. **(A)** Small intestine and mesentery encircling the mass, with proximal dilation (red arrow) and distal collapse (white arrow). Large blood vessels are visible on the mass surface (black arrow). **(B)** Gross pathology: solid gray-white mass with a gray-brown area. **(C)** Microscopy: spindle-shaped tumor cells with myxoid and collagenous areas, accompanied by significant inflammatory infiltration (hematoxylin and eosin staining, 10×).

The patient subsequently underwent surgical intervention. Intraoperatively, a mass, approximately 8.0 × 5.0 cm in size, was identified within the abdominal cavity (**Figures 4A**). The mass was firm and tightly adherent to the encasing small intestine and mesentery. Blood vessels supplying the mass were visible on the surface. The proximal bowel was dilated, and the distal bowel collapsed. Gross pathology of the specimen revealed a solid, gray-white, firm mass measuring 8.5 × 7.0 × 4.5 cm, with a gray-brown center (**Figure 4B**). Histopathological examination revealed proliferating spindle cells arranged in bundles accompanied by myxoid and collagenous areas with significant infiltration of lymphocytes, plasma cells, and foam cells (**Figure 4C**). Immunohistochemistry results were positive for ALK, CD68, SMA, STAT6 (focal), SDHB (localized), Ki-67 (20%), CD99, and Bcl-2, whereas markers such as h-CALD, S-100, CD34, Desmin, CD117, and Dog-1 were negative. The final pathological diagnosis confirmed an inflammatory myofibroblastic tumor in the small intestine, with both resected ends of the bowel showing negative margins. The patient recovered well postoperatively and was discharged. Six months later, routine ultrasonography revealed no signs of recurrence.

## Discussion

IMTs in children are rare neoplasms with low malignant potential. It predominantly occurs in the abdominopelvic cavity (including the mesentery, omentum, and retroperitoneum), followed by the lungs, head, neck, and extremities (2, 3, 6). Small-intestinal IMT is particularly rare and has mostly been reported in isolated cases (4, 5). The exact pathogenesis of IMT remains unclear; however, it may be associated with factors such as trauma, surgery, autoimmune diseases, and viral infections (2, 7). Genetic studies have identified various fusion genes, primarily receptor tyrosine kinase genes (ALK, ROS1, NTRK3, and PDGFRB), that may contribute to tumorigenesis (8). ALK gene rearrangement is the most common, occurring in approximately 50% to 60% of IMT cases (3, 8). Although IMT generally has a favorable prognosis, some patients may experience local recurrence, and rarely, distant metastases. Studies have shown that the local recurrence rate of extrapulmonary IMT is approximately 20%-25%, whereas that of distant metastasis can reach 7% (2, 3). Currently, surgical resection is the mainstay of treatment and long-term postoperative follow-up is necessary. For cases that are unresectable or metastatic, adjuvant therapies such as radiotherapy, chemotherapy, and targeted therapy have been used in some patients, although standardized treatment guidelines are yet to be established (2, 9).

Clinically, small intestinal IMT in children present insidiously with nonspecific symptoms, making early diagnosis challenging (4, 5). Some patients develop symptoms and complications due to tumors compressing the surrounding organs, commonly presenting with abdominal pain, vomiting, fever, hematochezia, and an abdominal mass (4, 6, 10). Laboratory tests may show elevated white blood cell counts and decreased hemoglobin levels; however, these features are not specific and usually normalize after complete tumor resection (4, 10, 11). In this case, the child presented with symptoms of bowel obstruction, including abdominal pain and vomiting, along with a mild elevation of inflammatory markers, microcytic hypochromic anemia, and thrombocytosis, consistent with previous reports (4, 10, 12). Abnormal laboratory findings in patients with IMT may be related to tumor-secreted inflammatory mediators, such as interleukin-6, interleukin-1b, and cyclin B1 (13, 14). Therefore, although elevated inflammatory markers, microcytic hypochromic anemia, and mild thrombocytosis may serve as auxiliary diagnostic indicators of small-intestinal IMT in children, their specificities are low. Histopathological examination and immunohistochemistry remain the gold standard for diagnosis, whereas imaging studies play a critical role in the initial assessment, preoperative diagnosis, and differential diagnosis.

The imaging features of IMT on CT or MRI are diverse and lack specificity, which may be related to the location of the lesion and variations in its fibrous and cellular components (13). Ultrasound is a safe, real-time, and repeatable imaging tool widely used in pediatric patients. Previous studies have shown that ultrasound has similar efficacy to CT and MRI in demonstrating the location, size, and boundaries of mesenteric IMT in children, as well as in assessing the relationship with surrounding tissues and the presence of distant abdominal metastases (15). Reports indicate that the ultrasound appearance of mesenteric IMT in children typically presents as a nodular or matted hypoechoic mass with heterogeneous internal echoes, vascular signals within the mass, and enhanced omental echoes surrounding the lesion (15). In this case, the small intestinal IMT appeared on ultrasound as a solitary, solid, hypoechoic mass with well-defined borders, heterogeneous internal echoes, and slightly hyperechoic fibrous strands without necrotic or cystic areas. Color Doppler ultrasound showed minimal blood flow signals within the mass originating from the wall and mesentery of the small intestine, which is consistent with previous reports (15). Additionally, ultrasound can be used to monitor real-time complications associated with intestinal tumors, such as bowel obstruction or intussusception. In this case, ultrasonography revealed proximal small intestinal dilation with normal peristalsis, suggesting partial small bowel obstruction, which was consistent with the findings of contrast-enhanced CT and intraoperative observations. In summary, the typical ultrasound appearance of a small intestinal IMT is a solitary, focal, hypoechoic mass with clear boundaries, slightly hyperechoic fibrous strands, minimal necrosis, and detectable blood flow signals within the tumor.

CEUS, an emerging imaging technique, provides significant advantages for early tumor diagnosis and differentiation by assessing microvascular perfusion. While conventional ultrasound may not clearly delineate the borders of larger lesions and Doppler ultrasound may sometimes fail to detect blood flow signals or identify necrotic areas, CEUS can overcome these limitations. Currently, there are limited reports on the combined use of ultrasound and CEUS for the diagnosis of IMT of the small intestine in children, with only a few case reports addressing other anatomical locations (16). The ultrasound contrast agent typically reaches the intestinal capillaries 10 to 20 seconds after injection, with peak concentration occurring between 30 and 40 seconds (17). According to the 2017 EFSUMB guidelines for CEUS, the arterial phase occurs within the first 30 seconds after contrast injection, reflecting the early blood supply to the tumor. The venous phase follows, occurring between 30 and 120 seconds, and is characterized by contrast washout, offering insights into tumor perfusion and vascularity. In this case, CEUS demonstrated synchronous enhancement of the lesion with the intestinal wall during the arterial phase, with a slightly hypo-enhancing pattern from the periphery to the center. The distribution of the contrast agent was relatively uniform, and washout was slow after reaching the peak. The lesion exhibited hypo enhancement during the venous phase and incomplete clearance in the late phase, similar to the persistent heterogeneous enhancement pattern observed in the delayed phase of contrast-enhanced CT. These characteristics may be related to the histological components of the lesion (8, 13). Coffin et al. pointed out that different IMTs or different areas within the same tumor may have variations in growth patterns and proportions of cells and stroma, which are primarily classified into three histological types: myxoid, spindle, and fibrous (18). Pathological examination revealed that the tumor was rich in spindles and inflammatory cells, with myxoid and collagenous areas. Therefore, the contrast agent penetrates the stroma through immature neovascularization within the tumor and is retained by the abundant collagen fibers and inflammatory cells outside the vessels, leading to slow washout of the contrast agent within the lesion (13). Additionally, some areas of the tumor exhibited marked echo attenuation on conventional ultrasound with no contrast enhancement on CEUS, which was likely due to the presence of abundant collagen fibers rather than necrotic areas, as confirmed by gross pathological and histological findings. Moreover, the peripheral enhancement observed during the arterial phase on CEUS, which is similar to the findings on contrast-enhanced CT, may be due to the presence of multiple feeding arteries encircling the tumor. Thus, we conclude that the CEUS characteristics of this pediatric small intestinal IMT include synchronous, centripetal, and slightly lower enhancement in the arterial phase with a slow washout after the peak, low enhancement in the venous phase, and no perfusion in dense collagenous areas.

In children, the differential diagnosis of small intestinal IMT requires distinction from other common small intestinal lesions (11). (i) Gastrointestinal stromal tumor (GIST): On ultrasonography, a GIST appears as an isoechoic or hypoechoic mass with heterogeneous internal echoes, often accompanied by necrosis and cystic changes with rich blood flow. CEUS typically shows centripetal heterogeneous enhancement with necrotic, non-perfused areas. (ii) Small intestinal lymphoma: Ultrasonography shows uneven thickening of the intestinal wall or solid infiltrative masses with extremely low internal echoes, rich blood supply, and enlarged peripheral lymph nodes. CEUS often demonstrates a uniformly high enhancement.

To confirm the diagnosis of IMT, histopathological and immunohistochemical examinations are essential (6, 13). Ki-67 is a reliable marker of cellular proliferation and reflects both the proliferative activity and malignant potential of tumor cells. Recent studies have indicated that the Ki-67 index is correlated with the prognosis of IMT. For instance, Song et al. (19) highlighted the importance of Ki-67 as a prognostic marker in a case of IMT from the greater omentum in children. Yuan et al. (20) reported that bladder IMT with a Ki-67 positivity of 15-20% is associated with a higher risk of recurrence. In our case, the Ki-67 positivity of 20% may suggests a higher risk of recurrence, which has important implications for clinical treatment and long-term follow-up decisions.

In summary, pediatric small intestinal IMT usually lack clear clinical features, making diagnosis difficult. Ultrasonography is the preferred imaging method for IMT diagnosis and postoperative follow-up because of its simplicity, speed, non-invasiveness, and repeatability. By accurately locating the lesion and evaluating its microvascular perfusion, CEUS helps determine the nature of the tumor and serves as an additional diagnostic tool. CEUS is recommended for the diagnosis of similar pediatric intestinal lesions. If mild hypo enhancement from the periphery to the center is observed during the arterial phase, followed by slow washout, small intestinal IMT should be considered.

## Data Availability

The original contributions presented in the study are included in the article/supplementary material. Further inquiries can be directed to the corresponding author/s.

## References

[1] Jo VY Fletcher CD . WHO classification of soft tissue tumours: an update based on the 2013 (4th) edition. Pathology. (2014) 46:95–104. doi: 10.1097/pat.0000000000000050 24378391

[2] Raitio A Losty PD . Treatment and outcomes in pediatric inflammatory myofibroblastic tumors - A systematic review of published studies. Eur J Surg Oncol. (2024) 50:108388. doi: 10.1016/j.ejso.2024.108388 38713995

[3] Rich BS Fishbein J Lautz T Rubalcava NS Kartal T Newman E . Inflammatory myofibroblastic tumor: A multi-institutional study from the Pediatric Surgical Oncology Research Collaborative. Int J Cancer. (2022) 151:1059–67. doi: 10.1002/ijc.34132 35604778

[4] Budylev A Solar I Kessner R Aizic A . ROS1-positive inflammatory myofibroblastic tumor of the small bowel causing obstruction: A case report. J Radiol Case Rep. (2022) 16:14–21. doi: 10.3941/jrcr.v16i1.3928 35586084 PMC9063829

[5] Oeconomopoulou A de Verney Y Kanavaki K Stefanaki K Pavlakis K Salakos C . Inflammatory myofibroblastic tumor of the small intestine mimicking acute appendicitis: a case report and review of the literature. J Med Case Rep. (2016) 10:100. doi: 10.1186/s13256-016-0880-0 27094797 PMC4837596

[6] Da M Qian B Mo X Xu C Wu H Jiang B . Inflammatory myofibroblastic tumors in children: A clinical retrospective study on 19 cases. Front Pediatr. (2021) 9:543078. doi: 10.3389/fped.2021.543078 34307241 PMC8295553

[7] Zhao J Han D Gao M Liu M Feng C Chen G . Inflammatory myofibroblastic tumor of the neck with thyroid invasion: a case report and literature review. Gland Surg. (2020) 9:1042–7. doi: 10.21037/gs-20-355 PMC7475359 32953613

[8] Preobrazhenskaya EV Suleymanova AM Bizin IV Zagrebin FA Romanko AA Saitova ES . Spectrum of kinase gene rearrangements in a large series of paediatric inflammatory myofibroblastic tumours. Histopathology. (2023) 83:109–15. doi: 10.1111/his.14912 37071060

[9] Craig E Wiltsie LM Beaupin LK Baig A Kozielski R Rothstein DH . Anaplastic lymphoma kinase inhibitor therapy in the treatment of inflammatory myofibroblastic tumors in pediatric patients: Case reports and literature review. J Pediatr Surg. (2021) 56:2364–71. doi: 10.1016/j.jpedsurg.2021.02.004 33676744

[10] Arredondo Montero J Pérez Riveros BP Bronte Anaut M Ros Briones R Bardají Pascual C . Ileal inflammatory myofibroblastic tumor in a two-month-old girl: long-term follow-up. Indian J Pediatr. (2022) 89:1264. doi: 10.1007/s12098-022-04367-x 36217007

[11] Kraus MS Selvam S Siddiqui I Reyes JA Chavhan GB . Imaging of pediatric gastrointestinal tumors: A tertiary center experience over 19 years. Eur J Radiol. (2024) 175:111461. doi: 10.1016/j.ejrad.2024.111461 38615503

[12] Zhao XT Yue SW Cheng Q Liu P Chang LY Zhao XX . CT findings of inflammatory myofibroblastic tumor of different pathological types. Zhonghua Yi Xue Za Zhi. (2017) 97:43–6. doi: 10.3760/cma.j.issn.0376-2491.2017.01.011 28056290

[13] Sargar KM Sheybani EF Shenoy A Aranake-Chrisinger J Khanna G . Pediatric fibroblastic and myofibroblastic tumors: A pictorial review. Radiographics. (2016) 36:1195–214. doi: 10.1148/rg.2016150191 27399243

[14] Mohamed AH Ahmed AT Al Abdulmonem W Bokov DO Shafie A Al-Hetty H . Interleukin-6 serves as a critical factor in various cancer progression and therapy. Med Oncol. (2024) 41:182. doi: 10.1007/s12032-024-02422-5 38900329

[15] Qian J Zhu K Ye J . Ultrasonic manifestations of mesenteric inflammatory myofibroblastic tumors in children. Front Pediatr. (2019) 7:39. doi: 10.3389/fped.2019.00039 30891434 PMC6411639

[16] Badea R Veres AA Andreica V Caraiani C Al-Hajjar N Sechel R . Inflammatory myofibroblastic tumor of the gallbladder: imaging aspects. J Med Ultrason. (2001). :89–95. doi: 10.1007/s10396-014-0566-4 26578495

[17] Sidhu PS Cantisani V Dietrich CF Gilja OH Saftoiu A Bartels E . The EFSUMB guidelines and recommendations for the clinical practice of contrast-enhanced ultrasound (CEUS) in non-hepatic applications: update 2017 (Long version). Ultraschall Med. (2018) 39:e2–e44. doi: 10.1055/a-0586-1107 29510439

[18] Coffin CM Watterson J Priest JR Dehner LP . Extrapulmonary inflammatory myofibroblastic tumor (inflammatory pseudotumor). A clinicopathologic and immunohistochemical study of 84 cases. Am J Surg Pathol. (1995) 19:859–72. doi: 10.1097/00000478-199508000-00001 7611533

[19] Song H Zhang H Zhang Y Wang X Liu W . Inflammatory myofibroblastic tumor from the greater omentum in children: A rare case report. J Cancer Res Ther. (2022) 18:2066–9. doi: 10.4103/jcrt.jcrt_1089_22 36647971

[20] Yuan H Wang Z Sun J Chu J Duan S Wang M . A rare huge bladder inflammatory myofibroblastic tumor treated by en bloc resection with diode laser: a case report and literature review. Front Oncol. (2024) 14:1327899. doi: 10.3389/fonc.2024.1327899 38529377 PMC10961466

